# Cell Behaviors during Closure of the Choroid Fissure in the Developing Eye

**DOI:** 10.3389/fncel.2018.00042

**Published:** 2018-02-20

**Authors:** Gaia Gestri, Naiara Bazin-Lopez, Clarissa Scholes, Stephen W. Wilson

**Affiliations:** Division of Biosciences, Department of Cell and Developmental Biology, University College London, London, United Kingdom

**Keywords:** eye, coloboma, choroid fissure, optic fissure, periocular mesenchyme, morphogenesis, optic vesicle, zebrafish

## Abstract

Coloboma is a defect in the morphogenesis of the eye that is a consequence of failure of choroid fissure fusion. It is among the most common congenital defects in humans and can significantly impact vision. However, very little is known about the cellular mechanisms that regulate choroid fissure closure. Using high-resolution confocal imaging of the zebrafish optic cup, we find that apico-basal polarity is re-modeled in cells lining the fissure in proximal to distal and inner to outer gradients during fusion. This process is accompanied by cell proliferation, displacement of vasculature, and contact between cells lining the choroid fissure and periocular mesenchyme (POM). To investigate the role of POM cells in closure of the fissure, we transplanted optic vesicles onto the yolk, allowing them to develop in a situation where they are depleted of POM. The choroid fissure forms normally in ectopic eyes but fusion fails in this condition, despite timely apposition of the nasal and temporal lips of the retina. This study resolves some of the cell behaviors underlying choroid fissure fusion and supports a role for POM in choroid fissure fusion.

## Introduction

In vertebrates, the eye forms through a series of coordinated interactions between tissues of different origins: the retinal neuroepithelium, non-neural surface ectoderm, and a loose array of cells arising from both neural crest and mesoderm known as periocular mesenchyme (POM; Fuhrmann, 2010). The primordium of the eyes, the eyefield, is specified as a single domain of cells in the anterior neural plate (Li et al., 1997; Zuber et al., 2003; Cavodeassi and Houart, 2012). Once specified, cells destined to form left and right eyes evaginate laterally, splitting the eyefield and giving rise to the optic vesicles, which evaginate laterally toward the surface ectoderm (Rembold et al., 2006; Kwan et al., 2012; Ivanovitch et al., 2013). Upon contact with the overlying ectoderm, the optic vesicle invaginates to form a double-layered optic cup; the inner layer is composed of prospective neural retinal cells and the outer layer is the primordium of retinal pigment epithelium (RPE; Chow and Lang, 2001; Martinez-Morales and Wittbrodt, 2009; Fuhrmann, 2010). This invagination process leads to the formation of a transient opening along the ventral retina and optic stalk termed the choroid, or optic, fissure (Schmitt and Dowling, 1994). Once the components of the POM that will give rise to the retinal vasculature have entered, and the retinal axons have exited the eye, the choroid fissure fuses (Gage et al., 2005; Hartsock et al., 2014; Williams and Bohnsack, 2015). Choroid fissure closure is a key event during eye development and failure of this process results in ocular colobomas, one of the most common hereditary ocular malformations that can profoundly affect vision (Onwochei et al., 2000; Morrison et al., 2002; Shah et al., 2011; Gestri et al., 2012).

Choroid fissure closure is considered different from other well-studied fusions (such as neural tube closure, wound healing and dorsal closure of Drosophila embryos), in which apical surfaces of opposing epithelial cells meet and fuse, a process that is characterized by dynamic apical protrusions that mediate initial cell-cell contacts (Gillian Morriss-Kay and Tuckett, 1985; Martin and Parkhurst, 2004; Tawk et al., 2007; Bazin-Lopez et al., 2015). By contrast, studies in rodents and fish have shown that the lips of the optic cup become apposed with intervening basal lamina, which must be degraded to allow the nasal and temporal retina to come into direct contact (Geeraets, 1976; Hero, 1989; James et al., 2016). Although it is the basal surfaces of retinal cells lining the fissure that approach each other during fusion, it is not known what happens to the polarity of these cells during the fusion process.

POM cells appear to play a critical role in ventral eye morphogenesis (reviewed in Gestri et al., 2012; Bazin-Lopez et al., 2015). Abrogation of genes encoding transcription factors implicated in POM development, such as *zic2, lmx1b*, and *TFAP2A/tfap2a* leads to lack of apposition of the ventral retinal lips and coloboma (Gestri et al., 2009; McMahon et al., 2009; Bassett et al., 2010; Lupo et al., 2011; Sedykh et al., 2017). However, these genes are expressed in other tissues that may affect eye morphogenesis, such as the lens placode and ventral diencephalon leaving the possibility that the observed ventral retinal phenotypes could be due to gene activity in domains other than the POM (Knight et al., 2003; Toyama et al., 2004; Hoffman et al., 2007; McMahon et al., 2009).

Retinoic acid (RA) signaling also contributes to ventral eye morphogenesis and choroid fissure fusion, acting both directly on the ventral optic cup, as well as regulating gene expression within the POM (Molotkov et al., 2006; Lupo et al., 2011). For instance, a late deficiency in retinoic acid prevents *pitx2* expression in the neural crest-derived POM and leads to coloboma (See and Clagett-Dame, 2009). Neural crest-specific knock-out of *Pitx2*, or all three retinoic acid receptor genes also causes abnormal ventral optic cup development and coloboma (Evans and Gage, 2005; Matt et al., 2008). Similarly, in humans, coloboma has been associated with neural crest defects in conditions such as CHARGE syndrome and frontonasal dysplasia (Siebert et al., 1985; Wu et al., 2007; Cordero et al., 2011).

POM ultimately contributes to numerous anterior segment and extraocular structures (Gage et al., 2005; Soules and Link, 2005) and some human patients show coloboma associated with anterior segment defects (Ozeki et al., 1999; Tang et al., 2006), again suggesting that coloboma can be linked to defective neural crest derived POM. However, it remains unclear how neural crest derived POM cells influence ventral eye morphogenesis and whether the coloboma phenotype is an inevitable secondary consequence of ventral eye morphogenesis defects that prevent any chance of apposition of the ventral retinal lips. Recently a role for mesodermal-POM in choroid fissure fusion has been investigated in zebrafish; mutants with both expanded and reduced hyaloid vasculature have coloboma (Weiss et al., 2012; James et al., 2016). On the other hand, fusion does occur in *cloche* mutants that lack ocular vasculature (Dhakal et al., 2015). This suggests that mesodermal-POM might promote but is not essential for choroid fissure fusion.

In this study, we use high-resolution 3D and 4D confocal imaging to analyze some of the key cellular events and behaviors that underlie choroid fissure fusion in zebrafish. We show that fusion is accompanied by basal lamina degradation and apico-basal remodeling of cells lining the fissure that results in the formation of an apical seam at the site of apposition. This seam retracts from the inner to outer retina to allow establishment of continuity of neuronal layers across the fusion site. By tracking single cells over time, we find that the cells lining the fissure are proliferative, although cell division appears not to be essential for fusion to proceed, and show numerous interactions with periocular mesenchymal cells. Supporting a role for POM cells in mediating choroid fissure fusion subsequent to apposition of the fissure lips, transplanted optic vesicles depleted of POM form normally shaped optic cups, but choroid fissures fail to fuse resulting in persistent coloboma.

## Materials and methods

### Animals

*AB* and *Tübingen* wild-type zebrafish strains, and transgenic lines, Tg(−7.2*sox10:*e-GFP)^zf77^ (Hoffman et al., 2007); Tg(–5 kb *lmx1b.1*:GFP)^mw11^ (McMahon et al., 2009); Tg(*Bactin*:HRAS-EGFP)^vu119^(Cooper et al., 2005); Tg(*fli1a*:EGFP)^y5^ (Lawson and Weinstein, 2002); Tg (ctnna-citrine)^ct3aGT^ (Žigman et al., 2011); NO067^*t*3071^ (Rossi et al., 2015), were maintained and bred according to standard procedures (Westerfield, 1995). Ethical approval for zebrafish experiments was obtained from the Home Office UK under the Animal Scientific Procedures Act 1986.

### Microinjection and cell transplantations

Donor embryos at the 1 cell stage were injected with mRNA encoding cytoplasmic-GFP or membrane-Cherry Fluorescent Protein. At the mid-blastula stage around 5–20 cells from the apical region of the donor embryo were transplanted to the same region in the stage-matched host. Cell transplantation was performed as previously described (Cavodeassi et al., 2005).

### Imaging and data processing

Embryos were mounted in 1% low melting agarose gel in embryo medium. They were imaged from a lateral angle so that the fissure was visible using a Leica SP8 microscope with at 25x water immersion lens in a chamber heated to 28°C. Z-stacks were acquired every 5–20 min for up to 12 h. Timelapse movies were analyzed using Volocity and Imaris software.

### Immunohistochemistry

Whole-mount immunolabeling procedures were performed as previously described (Wilson et al., 1990). For antibody staining of cryosections, embryos were first protected by sequential incubation in 15% then 30% sucrose in phosphate-buffered saline supplemented with 0.5% Triton X-100 (PBST) for 30′, embedded in OCT, stored at −80°C, and sectioned at 18 μm using a Leica cryostat. Primary antibodies were as follows: mouse anti-*zonula occludens* 1 (ZO1; 1:600, Sigma), rabbit anti-laminin (1:600, Sigma), chicken anti-GFP (1:1,000; Sigma). The secondary antibodies were: Alexa Fluor 633 anti-mouse, 488 anti-rabbit, and 488 anti-chicken (all 1:1,000, Invitrogen). Images were collected on a Leica confocal microscope using a 40x oil immersion lens. Gain and offset were adjusted to enhance the contrast of the signal against the background.

### Histology

Sectioning was as for immunohistochemistry; host embryos were oriented such that sagittal sections would be cut through the transplanted eye. To visualize retinal organization, slides were dipped in the nuclear marker methylene blue (0.033%) for 90 s and imaged while wet without cover-slipping.

### TUNEL analysis

To detect apoptotic cells, TUNEL labeling was carried out using the Apoptag kit (Chemicon International).

### Blocking cell division

To block cell division, embryos were cultured in embryo medium containing 100 μM aphidicolin and 20 mM hydroxyurea dissolved in 2% dimethylsulphoxide from 36 to 60 hpf (Tawk et al., 2007).

### Optic vesicle transplants

Transplantation of optic vesicles to the yolk was performed as described by Picker and Brand (2005). We used Tg(−7.2*sox10:*e-GFP)^zf77^ (Hoffman et al., 2007) embryos as hosts and donors. Donors were injected at 1–4-cell stage with RNA encoding membrane-RFP. Stage-matched donor and host embryos were dechorionated in E3 on an agar plate. Embryos were staged and then mounted in 1.3% low-melt agarose (LMA) in E3 at 42°C on culture dishes. Once the agarose had solidified, Ringer's solution + 1/250 P+S antibiotic was added to cover the agarose droplet, and a flap removed to expose eye or yolk (in the case of donor or host, respectively). A droplet of mineral oil (Sigma M-8410) was micro-pipetted onto the skin behind and in front of eye and left for 5 min to dissolve the ectoderm. The ectoderm was pierced with a sharpened tungsten needle and the optic vesicle cut away from forebrain. The time of this cut was recorded to calculate stage at which transplantation occurred. A hole was made in the ectoderm of yolk using mineral oil, and widened. The optic vesicle was removed from the donor, by cutting away its surrounding ectoderm, and transferred to host embryo using a tungsten wire loop. Once placed into the hole it was pushed under the ectoderm of the yolk. The donor was then cut loose from the agarose, staged, and fixed immediately in 4% PFA. Transplants were left to develop at 28.5°C. A few hours after transplantation, the host embryo was removed from its agarose and transferred to E3+PTU. The transplant was live-imaged in 1% LMA under a Leica confocal microscope with a 40x water immersion lens. This was done between 2 and 5 h post-transplant (hpt) and at 34 hpf. Bright field imaging was carried out using a Nikon eclipse E1000 microscope at 10x or 20x magnification. Experimental embryos were then grown to 5 dpf at 28.5°C and fixed in 4% PFA at 4°C.

## Results

### Choroid fissure cells reorient their apico-basal axes as fusion progresses both along proximo-distal and inner to outer retinal axes

**Figure 1:**
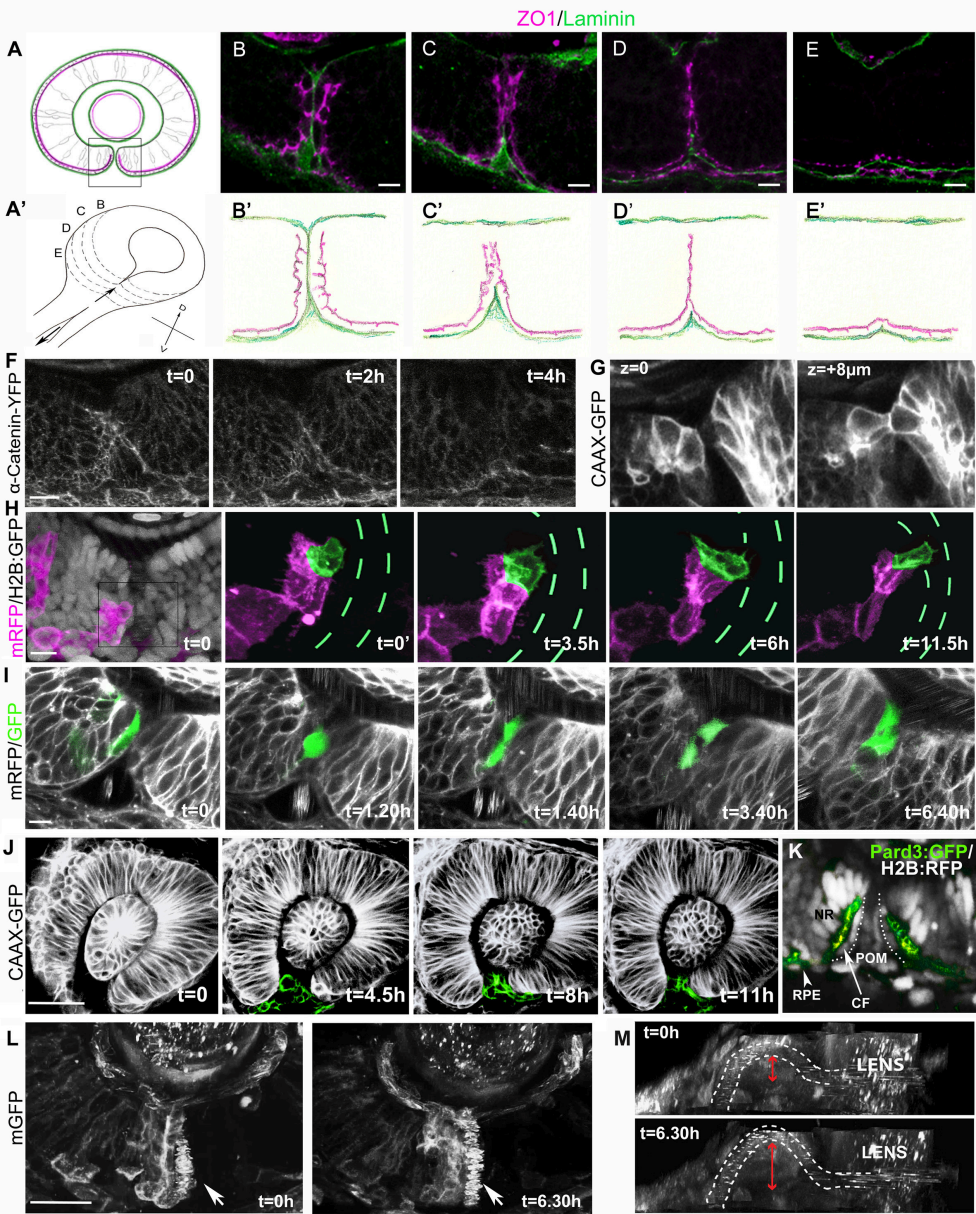
Cell organization and behavior during choroid fissure fusion. **(A)** Schematic showing the optic cup in parasagittal section at the level of the lens just prior to choroid fissure fusion. The cells lining the fissure margins are continuous with the retinal pigmented epithelium and approach each other with their basal surfaces (green) facing the fissure and their apical surfaces (magenta) at the sub-retinal space (ventricular lumen of the optic vesicle). Boxed region corresponds to retinal regions shown in **(B–I)**. Dorsal is up. **(A')** Schematic view of the optic cup midway through choroid fissure fusion. Letters **(B–E)** correspond to the positions of schematics/sections in **(B–E, B'–E')**. **(B–E)** Sections through the choroid fissure during fusion showing apically-located zonula occludens labeling (ZO1; magenta) and the basal lamina component Laminin (green) in the ventral retina of a 44 hpf embryo. **(B'–E')** Schematic of progressive steps in choroid fissure fusion process based on analysis of eyes from 8 embryos at 44 hpf (as in **B–E**). **(F–L)** Various views of the closing choroid fissure with transgenic lines analyzed or protein derived from injected RNA indicated to the left of the panels. **(F)** Image stills over a 4 h period from a 10 h time-lapse movie (Movie S1) from 34 to 44 hpf of Tg(*ctnna*:citrine)^ct3aGT^ labeling of α-catenin-labeled adherens junctions in the fusing choroid fissure. **(G)** Z-stack images at two depths of the choroid fissure showing cell shapes just prior to and at the point of fusion in a 44 hpf eye. **(H)** Image stills over 11.5 h from a time lapse movie (Movie S3) from 34 to 45.5 hpf showing transplanted cells expressing mRFP (magenta) in an eye in which all cells are expressing nuclear H2B-GFP (gray in t = 0); one cell located in the fissure has been pseudocolored in green and the H2B-GFP fluorescence removed from the four panels on the right. Green dashes highlight the position of the fissure (visible when viewing H2B-GFP expression). **(I)** Image stills over 6 h 40 m from a 12 h time lapse movie (Movie S4) from 34 to 46 hpf showing transplanted cells in the fissure expressing cytoplasmic-GFP in an eye expressing mRFP. The fissure cell divides once during the course of the movie. **(J)** Image stills over 11 h from a 12 h time lapse movie (Movie S5) from 26 to 38 hpf showing POM cells expressing CAAX-GFP (pseudocolored in green). **(K)** Image of the choroid fissure prior to fusion showing apically-located GFP-tagged Pard3 labeling (green) and nuclear staining (gray) in the ventral retina of a 40 hpf embryo. The image shows that cells lining the choroid fissure (CF) are in continuity with the retinal pigment epithelium (RPE) and the pseudo-stratified/stratified neural retina (NR). **(L,M)** Image stills over 6.5 h from a 12 h time lapse movie (Movie S6) from 34 to 46 hpf showing the superficial displacement of the hyaloid vessel during choroid fissure closure. The orientation in K is as other panels above; in L, the image has been rotated to give a lateral view into the fissure; the irregular spots inside the white dashed lines (outlining the hyaloid vessel) are a result of movement of blood cells. The superficial displacement of the vessel is shown by the red arrow. Scale bars: **(B–I)** ~10 μM; **(J,K)** ~30 μM.

In zebrafish, choroid fissure closure initiates at around 30 hpf and spreads bi-directionally in a zipper-like manner, distally along the ventral retina and proximally along the optic stalk until fusion is complete by 56 hpf (Figures 1A'–E'; James et al., 2016 and data not shown). In most fusion events, it is the apical surfaces of the epithelia that approach each other and initiate fusion. However, it is the basal epithelial surfaces that approach each other as the choroid fissure closes. Choroid fissure cells are part of a continuous epithelium bridging between the squamous cells of the retinal pigment epithelium and the pseudo-stratified/stratified neural retina (Hero, 1989; Figure 1K); the apical surfaces of these epithelia contact the narrow ventricle that later forms the sub-retinal space between RPE and photoreceptors; the basal surfaces of the epithelia contact the basal lamina continuous from the outside of the eye, through the fissure to the inner surface of the retina. As fusion happens, the basal lamina within the fissure must disappear, the continuity of the epithelium from RPE through the fissure to neural retina must break down, and new connections must form between both the neural retinal cells and the RPE cells initially on nasal and temporal sides of the fissure. The cellular events that occur during these events are not understood. To begin to characterize the remodeling of the cells within the fissure, we examined the distribution of zonula occludens-1 (ZO1) which localizes at apical functional complexes, and Laminin, a main component of the basal lamina beneath the basal epithelial surface.

Imaging of ZO1 labeling showed that although fissure cells initially approach each other through their basal surfaces, there is apical apposition of nasal and temporal cells at the point of fusion. At 44 hpf the fissure is part way through closure and so serial parasagittal sections through the eye capture all stages of the fusion process (Figures 1A–E'). Just prior to fusion, the basal lamina lining nasal and temporal lips of the fissure has reduced to a single line of Laminin labeling, and although apical ZO1 labeling is becoming somewhat disorganized, it is largely absent from the cell surfaces contacting the Laminin (Figures 1B,B'). However, by the stage that Laminin immunoreactivity has been lost from the fissure, there is just a single stripe of ZO1 labeling along the remnants of the fissure; this implies that the apical surfaces of cells lining nasal and temporal lips of the fissure cells must be in close apposition (Figures 1D,D'). As fusion completes, all ZO1 labeling retracts progressively from the inner to the outer retina adjacent to the sub-retinal space (Figures 1D–E'). This retraction is also evident through live imaging of α-catenin-labeled adherens junctions (Tg(*ctnna*:citrine)^ct3aGT^, (Žigman et al., 2011) which shows the disappearance of apical markers over a period of about 4 h during fusion (Figure 1F, Movie S1; *n* = 1 movie of 10 h). We have not resolved the eventual fate of the cells lining the fissure, but the retraction described above suggests some such cells may move toward the outer retina and join the RPE. However, from other movies, nuclear tracking suggests that some cells lining the fissure may actually move toward the inner retinal surface where they could incorporate into the neural retina (Movie S2). Resolution of this issue will require tracking of the fissure lining cells (3–5 cells along each lip of the fissure from inner to outer retina) for longer periods of time.

### Fissure cells have cuboidal morphology and show protrusive activity

Visualization of GFP-labeled cells in Tg(β-*actin*:HRAS-EGFP)^vu119^ embryos (Cooper et al., 2005) showed that prior to and during fusion, retinal cells lining the fissure have a cuboidal morphology, intermediate between the columnar cells of the neural retina and the squamous cells of the retinal pigmented epithelium (Figure 1G, two Z-stacks 8 μm apart). To characterize the behaviors of these cells during the tissue remodeling that accompanies fusion, we mosaically labeled cells lining the fissure by transplantation of donor mRFP expressing cells into host blastulae expressing nuclear-targeted GFP, and used high-resolution 4D confocal imaging to observe cell behaviors (5 cells imaged within the fissure).

Figure 1H and Movie S3 show an example of a fissure cell (green) that is cuboidal in shape with epithelial character at the onset of imaging (*t* = 0). After 3.5 h, protrusive activity is evident but without a clear directionality. However, from 6 h, the cell is more overtly polarized toward the temporal lip of the fissure, and displays protrusive activity in the vicinity of cells in the fissure with very motile nuclei that we presume belong to the POM. These protrusions could potentially be involved in the formation of new retinal cell-cell contacts during choroid fissure fusion.

### Cells lining the fissure frequently undergo cell division during fusion

In cell transplantation experiments, we observed that 17 out of 19 GFP positive cells targeted to the fissure divided at least once during timelapse acquisition (six movies, each over a period of 8/12 h; Figure 1I, Movie S4). Although neural retina cells are still proliferative at this stage, RPE cells are thought to have exited the cell cycle. This suggests that while cells lining the fissure and the RPE are part of a contiguous epithelium their behaviors are different.

Cell cycle progression regulates neural crest cell delamination from the neural tube (Burstyn-Cohen and Kalcheim, 2002) and cell proliferation can be a key player driving epithelial morphogenesis (Kondo and Hayashi, 2013). To test whether cell proliferation is required for epithelial remodeling during choroid fissure fusion, embryos were bathed in hydroxyurea and aphidicolin (HUA) from 30 to 60 hpf to block cell division (Harris and Hartenstein, 1991). The eyes of HUA treated embryos are smaller, but Laminin and ZO1 immunoreactivity was almost always absent within the optic cup at the position where the fissure lips meet and fuse (90%, *n* = 120) as in wild-types (100%, *n* = 80), suggesting that cell proliferation is not essential for fissure fusion (data not shown).

### Cell death is not required for fissure fusion

**Figure 2:**
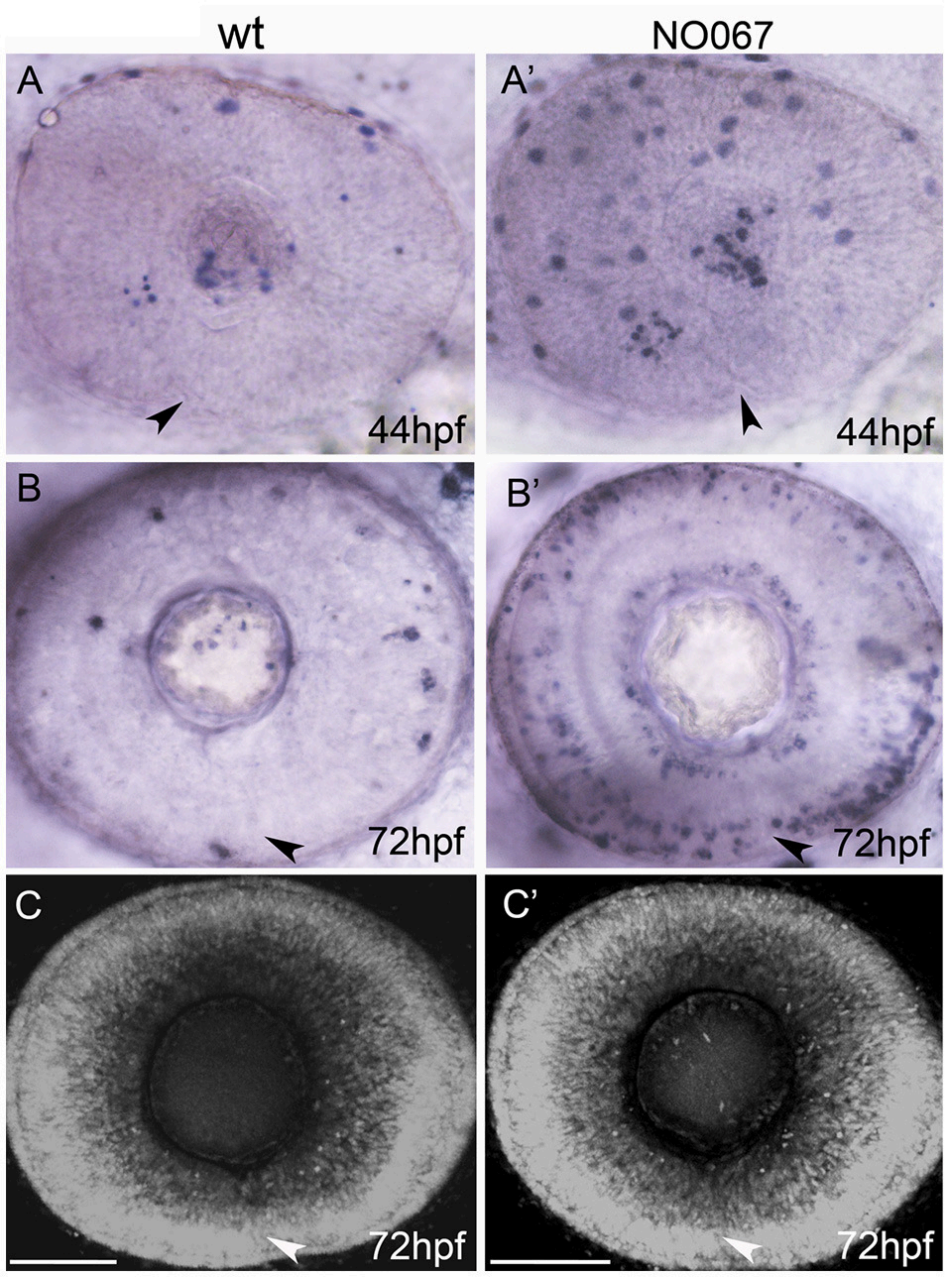
Cell death is not required for choroid fissure fusion. Images of whole eyes **(A–B')** or Z-stacks of the retina **(C–C')** of wildtype (left column) and NO067 mutant (right column) embryos at ages shown bottom right. Arrows show position of the choroid fissure. **(A–B')** TUNEL labeling (blue) of dying cells. There are more apoptotic cells in the mutant eye but they do not localize to the choroid fissure. **(C–C')** DAPI staining of nuclei showing the retina of the mutant eye has undergone normal morphogenesis in the absence of macrophages. Scale bar: 50 μM.

In mouse and hamster, apoptosis has been reported to occur in the fissure margins during fusion, suggesting that cell death might promote basal lamina breakdown and retinal fusion (Geeraets, 1976; Hero, 1989). To assess cell death in the choroid fissure in zebrafish, TUNEL staining was carried out at various stages during fissure fusion. No cell death specifically localized to the fissure was observed at 40, 44, 48, and 56 hpf (*n* = 20 embryos per stage, data not shown; James et al., 2016). In addition, no cell death was observed in the fissure during *in vivo* imaging of the ventral eye in Tg(β-*actin*:HRAS-EGFP)^vu119^ and Tg(*ctnna*:citrine)^ct3aGT^ embryos and embryos with mosaically GFP-labeled cells lining the fissure (Figures 1F,H–J). Macrophages are abundant during eye and brain development (Herbomel et al., 1999) and moreover macrophages were occasionally observed around the choroid fissure in various time-lapse movies (data not shown); this raises the possibility that the lack of an overt cell death might be due to efficient removal of dying cells. In order to address if this may be the case, TUNEL analysis and fusion was analyzed in NO067^*t*30713^ mutant embryos that lack macrophages in the eyes and brain (Rossi et al., 2015). While an increase in cell death was observed in the RPE of mutants at 44 hpf (compare Figure 2A with Figure 2A') and in different layers of the neural retina at 72 hpf (compare Figure 2B with Figure 2B'), no significant increase in cell death was observed in the choroid fissure which closed effectively in mutants (compare Figures 2B,C with Figures 2B',C'; 18 eyes per stage were analyzed and TUNEL positive cells in the choroid fissure counted). These observations suggest that cell death is not required for the choroid fissure to close in zebrafish.

### Motile POM cells are present in the fissure up to the point of fusion

Prior to closure, the choroid fissure allows POM cells of both neural crest and mesodermal origin to migrate inside the eye (Gestri et al., 2012). To visualize POM cell behaviors during fusion we imaged GFP-labeled neural crest cells in Tg(−5kb *lmx1b.1*:GFP)^mw11^ embryos (McMahon et al., 2009; data not shown) as well as all POM cells in the fissure in (Tg(β-*actin*:HRAS-EGFP)^vu119^ embryos in which membrane targeted GFP is expressed by all cells (Cooper et al., 2005).

Just prior to closure there are highly motile POM cells within the fissure. POM cells extend processes to contact retinal cells in both nasal and temporal lips of the fissure during fusion (Figure 1J from Movie S5 which is one of three movies over periods of 14 h, and data not shown). Similar interactions between choroid fissure cells and the mesodermal component of the POM that will give rise to the vasculature also occurs (James et al., 2016; Eckert et al., in review and data not shown). The zippering closure of the fissure at the level of the optic cup results in the superficial displacement of the prospective hyaloid artery to the ventral surface of the retina (Figures 1L,M; Movie S6).

Despite the presence of mesodermally-derived POM in the choroid fissure, mutants that likely miss such cells, such as *cloche* (Stainier et al., 1995), appear to have normally closed fissures by 60 hpf, even if a delay in fusion has been reported (James et al., 2016 and data not shown). In contrast, loss of function of genes that are expressed in the neural crest-derived POM or throughout the POM present forebrain and eye morphogenesis defects associated with coloboma (Gage et al., 1999; Gestri et al., 2009; McMahon et al., 2009; Bassett et al., 2010; Sedykh et al., 2017).

### Optic cup morphogenesis is normal, but choroid fissue fusion fails in transplanted optic vesicles with depleted POM

As the optic vesicle evaginates, neural crest derived POM cells progressively migrate over and around the forming eye (Figure S1C, Langenberg et al., 2008). Thus, to assess whether neural crest cells contribute to proper eye morphogenesis and choroid fissure fusion, in a condition in which the rest of the brain is not affected, we isolated optic vesicles prior to the completion of neural crest migration over the vesicle and transplanted them onto the yolk of host embryos (Picker and Brand, 2005; Figure S1 and Methods for details). When donor optic vesicles were transplanted orthotopically in host embryos, in place of endogenous eyes, they develop normally and choroid fissure fusion occurs (Figures S1F,G). For technical reasons, we were unable to extirpate the optic vesicles prior to arrival of any neural crest cells and so all transplants included some neural crest cells (see Figure S1H, Movies S7, S8).

**Figure 3:**
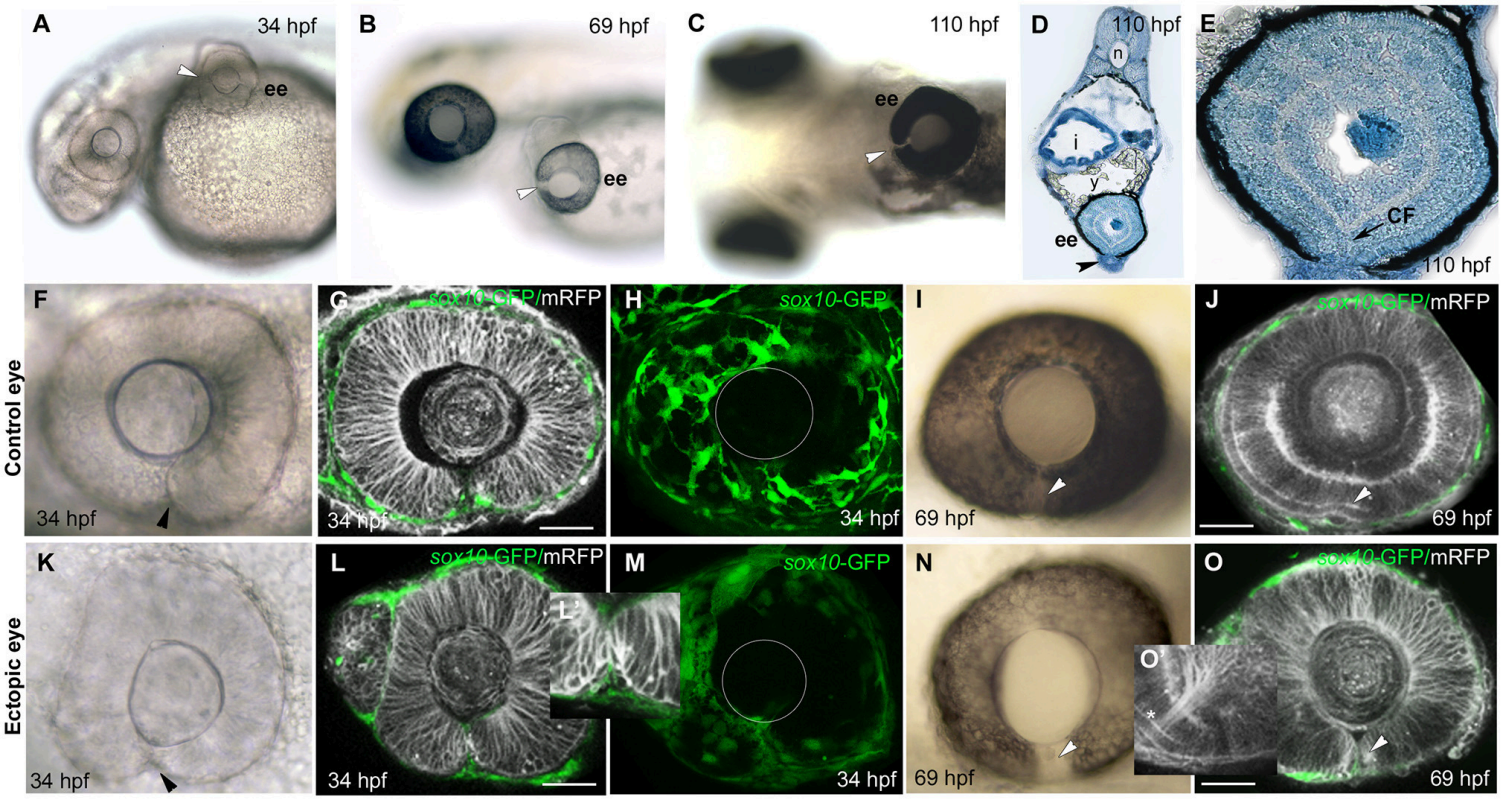
Failure of choroid fissure fusion in eyes derived from optic vesicles with reduced POM. **(A–C)** Brightfield images of two host embryos at different stages after optic vesicle transplantation (at 12 ss, about 14 h pf), showing location of the ectopic eye on the yolk. Arrowheads indicate the open choroid fissure in the ectopic eyes. **(D,E)** Methylene blue stained sagittal sections at low **(D)** and high **(E)** resolution through an ectopic eye at 5 dpf. **(F–O)** Images of control **(F–J)** and ectopic **(K–O)** eyes imaged either in brightfield **(F,K,I,N)** or by confocal microscopy **(G,H,L,M,J,O)** showing cell membranes (mRFP, gray) and *sox10*:GFP labeled POM (green) at ages shown on the panels. Arrowheads indicate the choroid fissure. Asterisk in O' indicates retinal ganglion cell axons. The position of the lens is indicated with a white circle in **(H)** and **(M)**. ee, ectopic eye; i, intestine; sc, spinal cord; y, yolk. Scale bars 50 μM.

Ectopic optic vesicles underwent overtly normal morphogenesis to form isolated optic cups/eyes on the yolk (Figure 3; *n* = 12 transplants of 12–18 ss optic vesicles). The ectopic optic vesicles had variably reduced amounts of Tg(-7.2*sox10*;eGFP)^zf77^ labeled neural crest cells (compare control eye in Figure 3H with transplanted eye Figure 3M and Figure S1H). Furthermore, no recruitment of host neural crest was observed in 4 out of 5 transplants analyzed (*n* = 5 mRFP expressing optic vesicles transplanted in Tg(*sox10*:GFP^zf77^) positive hosts; Movies S7, S8). Furthermore, the lack of hyaloid space between the lens and the retina in ectopic eyes suggests an absence of vasculature (Figures 3G,L and data not shown). Thus, in ectopic eyes it is likely that the mesodermal component of the POM is also considerably reduced or absent.

Despite the lack of full coverage of optic vesicles by POM, they always invaginated properly to form bi-layered optic cups that were virtually indistinguishable from host eyes (compare Figures 3K,L with Figures 3F,G). The choroid fissure formed in ectopic eyes and its lips came into close apposition (Figures 3K,L,L'). The only overt difference in ectopic eyes was the reduction in hyaloid space between the retina and the lens (Figures 3F,G,K,L; Gage et al., 2005). By 70 hpf, the photoreceptor layer was visible in the neural retina of ectopic eyes and RGC axons had coalesced at the optic nerve head and exited the eye (Figures 3J,O, asterisk in O').

Despite the overtly normal development of ectopic eyes over 5 days of development, choroid fissures failed to fuse (Figures 3B,C,N). To better assess the choroid fissure phenotype at 5 dpf, we sectioned ectopic eyes in the sagittal plane (*n* = 4; Figures 3D,E and Figure S2). In control eyes, by 60 hpf there is a continuity of both neural retina and RPE all around the eye (Figure S2 and data not shown). The ectopic eyes showed well-developed lamination of the retina, with the outer and inner nuclear layers, inner plexiform layer and ganglion cell layer all clearly visible, but there was disjunction between nasal and temporal retinal layers, most obvious in the discontinuity of the RPE (Figures 3D,E). These are features indicative of persistent coloboma.

We noticed that the lack of fusion is most evident superficially within the retina (Figures S2D,E), but in deeper sections of the ectopic eyes there was continuity in the photoreceptor layer (*n* = 2; Figures S2C,F). Thus, it is possible that the most deep layer of the choroid fissure can fuse in ectopic eyes or an alternative possibility is that it may not have been fully induced to form normally. Indeed when vesicles removed for transplantation at slightly earlier stages (10/12 ss), no choroid fissure formed in the ectopic eye at 24 hpf, suggesting that fissure specification may not be complete by 10/12 ss (*n* = 2; data not shown).

These analyses show that morphogenesis of the optic cup can occur normally in transplanted vesicles in the presence of reduced amounts of POM cells, and that the lips of the choroid fissure come into apposition in a timely manner. Nonetheless, fissure fusion fails to occur by 5 dpf, leaving the transplant eyes displaying a classic coloboma phenotype.

## Discussion

In this study, we provide both a detailed description of many of the cellular events that accompany choroid fissure closure in zebrafish and evidence consistent with a role for POM in mediating fusion subsequent to apposition of the lips of the fissure. We show that although cells lining the fissure approach each other via their basal surfaces, at the point of fusion, it appears that there is transient apical to apical cell contact. Many fissure cells undergo cell division but few, if any, die during fusion and neither proliferation nor cell death appears necessary for fusion to occur. We show that optic vesicles depleted of POM develop into normally shaped optic cups with well-formed choroid fissures. However, choroid fissures in ectopic eyes fail to fuse indicating that the local environment in which the eye forms impacts choroid fissure fusion. This would be consistent with a normal complement of POM being required for choroid fissure fusion.

### Choroid fissure lip cells repolarize during fusion

Most epithelial fusion events are initiated by apical to apical contacts between the cells undergoing fusion (Jacinto et al., 2001). Choroid fissure fusion has been considered unusual in this respect in that the two epithelia destined to fuse approach basal surface to basal surface with two intervening basal laminae. Our data suggests that choroid fissure fusion may not be so different to other epithelial fusions as the initial apico-basal polarity of fissure cells breaks down and reorganizes such that at the point of fusion, there is an apical seam at the interface between fusing cells. We assume that fissure lip cells contribute to making this apical seam, but an alternative possibility is that neural retinal cells could displace the lip cells at the point of fusion (although we have not observed the dispersal of fissure lip cells that would happen if this was the case). Resolution of this issue will require mosaic labeling of fissure lining cells coupled with long-duration imaging to resolve their eventual fates.

As they approach each other, we find that cells lining the nasal and temporal lips of the choroid fissure have cuboidal morphology distinct from both the RPE and neural retina. Indeed several lines of evidence suggest that fissure cells are a distinct population. Not only does their morphology and behavior differ from their neighboring retinal cells, they also have a distinct gene expression profile; for example *netrin1a* and *integrina5* are only expressed in cells lining the fissure (Lupo et al., 2011 and references within). A somewhat fluid identity of the fissure cells is suggested by the observation that they lack *rx2* expression prior to fusion (similar to RPE cells) whereas they express *rx2* after fusion (as do neural retinal cells; Eckert et al., in review), on the other hand, they remain proliferative while most other RPE cells have exited the cell cycle (Hu and Easter, 1999; Cechmanek and McFarlane, 2017).

The mechanisms by which cells lining the fissure break down their initial apico-basal polarity remain to be determined but it is likely that the loss of basal lamina is a key trigger for this event. In other situations loss of basal lamina can lead to epithelial cells reorienting their apico-basal polarity. For instance, in the early evaginating optic vesicle in zebrafish, disrupting Laminin can lead to complete inversion of cell polarity such that apical proteins appear at the basal surface of the optic vesicle and cells adopt a more mesenchymal morphology (Ivanovitch et al., 2013). Our results also suggest that although epithelial apico-basal polarity must inevitably be re-modeled during the cell divisions that fissure cells undergo, this appears not to be a prerequisite for successful fusion.

### Choroid fissures form in isolated optic cups

Young optic vesicles depleted of POM and transplanted heterotopically onto the yolk developed into optic cups/eyes almost indistinguishable from wild-type eyes. Notably, optic cup morphogenesis appeared normal, a choroid fissure formed and the nasal and temporal margins of the fissure became properly apposed. Consequently many aspects of eye formation can occur in retinal cells isolated from their normal environment. This is demonstrated perhaps most dramatically in the ability of ES cell organoids to form optic cups that contain differentiated RPE cells and retinal neurons (Eiraku et al., 2011). Indeed, more recent studies have demonstrated that retinal organoids can even acquire dorso-ventral polarity and form clefts resembling choroid fissures (Hasegawa et al., 2016).

The inductive events leading to choroid fissure formation are poorly understood although there appears to be a requirement for BMP7 signaling and in retinal organoids, BMP signaling enhances DV polarization of optic cups (and consequently fissure formation; Morcillo et al., 2006; Hasegawa et al., 2016). Optic vesicle rotation experiments in chick have defined the stage at which fissure specification has occurred. Comparable preliminary experiments in fish (unpublished data) suggest fissure specification may occur between 9 and 12 ss, subsequent to the allocation of naso-temporal identity that is needed to define the position of the fissure (Picker et al., 2009; Hernández-Bejarano et al., 2015). Indeed in two early stage (12 ss) optic vesicle transplants, the fissure did not span the entire depth of the optic cup. These observations suggest that for all but the earliest optic vesicle transplants, specification of the fissure had occurred prior to extirpation and that subsequent formation of the fissure is an intrinsic property of the optic vesicle/cup.

### Choroid fissures fail to close in isolated optic cups

A striking observation was that although morphogenesis proceeded normally in isolated optic cups, the apposed lips of the choroid fissure always failed to fuse. Experiments in which eye primordia were transplanted ectopically in chick showed similar results (Gayer, 1942). This suggests that cells/signals present in the normal environment in which the eye forms and absent/reduced in isolated optic vesicles are required for fissure fusion. The isolated optic cups are separated from the optic stalk/ventral forebrain and are depleted of POM and both cell populations are candidates as being involved in mediating fusion. Perhaps arguing against a significant role for late signals from ventral forebrain/optic stalk is our observation that optic vesicles transplanted orthotopically show fissure fusion despite severing the link between optic vesicle and brain. However, we did not assess if such connections reformed and it remains possible that signals from ventral forebrain could still influence the choroid fissure in orthotopic transplanted optic cups.

There is considerably more evidence, albeit mostly circumstantial, that POM influences fissure fusion. Indeed, in this study we find that highly motile POM cells elaborate processes that extend and contact cells on both sides of the fissure right up to the point of fusion. The POM is composed of both mesodermal and neural crest components and either or both cell populations could regulate fissure fusion. A notable absence from the ectopic eyes is the hyaloid vasculature, which normally enters the optic cup though the choroid fissure. The vasculature forms just prior to fissure fusion, with contribution from both components of the POM (Isogai et al., 2001; Gage et al., 2005). The mesodermal POM contributes the vascular endothelial cells while the neural crest POM provides the pericytes of the vessel walls (Etchevers et al., 2001; Gage et al., 2005). Since a key role for the choroid fissure is to allow access of circulation to the developing lens and retina, it would make sense for there to be a mechanism to ensure that fusion is not initiated until the hyaloid vasculature is in place. Indeed, fusion tends to commence from the level of the optic disk, where the vasculature enters the eye, and proceeds proximally and distally from this point (Geeraets, 1976; Hero, 1989; James et al., 2016; Figure 1). One possibility is that the forming hyaloid vessels are the source of matrix metalloproteinases (Heissig et al., 2003; Lafleur et al., 2003) that assist in basal lamina dissolution within the fissure margins. This said, choroid fissure fusion still occurs in zebrafish lines carrying mutations in genes required for cephalic vascular development, such as *cloche* (Stainier et al., 1995; Dhakal et al., 2015). This suggests that even if the mesodermal POM/vasculature plays a role in fusion, it may not be essential, perhaps redundant with comparable activity from neural-crest derived POM.

There is considerable evidence that disrupting neural-crest derived POM can lead to optic vesicle morphogenesis defects and coloboma (Bazin-Lopez et al., 2015). However, conclusions on the role of neural crest POM in early eye development are not clearcut; indeed loss of function of some genes impacting neural crest derived-POM, such as *Foxc1* in mouse, does not appear to lead to coloboma and *Foxc1* mutations in humans are only rarely associated with coloboma (Kidson et al., 1999; Kaur et al., 2009; Tümer and Bach-Holm, 2009). Furthermore, disrupting genes expressed in the neural crest (and often elsewhere) can lead to craniofacial deficits and problems in optic vesicle morphogenesis so severe that the nasal and temporal lips of the fissure are never close to being in apposition. Coloboma in such situations is a cosequence of mechanistically very different events than those that fail when the fissure forms normally, its lips appose but there is a failure to fuse. As yet there is little direct evidence for a role for the neural-crest derived POM in mediating the final steps of fusion. Nevertheless, our data is entirely consistent with such a role and indeed suggests that depletion of both components of the POM may lead to failure of fusion, bypassing the possibility of functional compensation when either mesodermal or neural crest-derived POM alone is affected. We consider that it remains an attractive possibility that POM in the fissure contributes to, or is required for, the breakdown of the basal lamina, enabling direct contact between the two opposing lips of the fissure.

### POM may not be required for optic cup morphogenesis

We show that optic cups form relatively normally when depleted of POM comparable to the situation when optic cups form in ES cell organoids (Eiraku et al., 2011; Hasegawa et al., 2016). This contrasts with many situations *in vivo* in zebrafish, mice, and humans in which genes that impact POM development are disrupted and eyes exhibit very severe morphogenesis defects (Gage et al., 1999; Gestri et al., 2009; McMahon et al., 2009; Bassett et al., 2010; Sedykh et al., 2017). We cannot rule out the possibility that the reduced numbers of POM cells carried with the transplanted optic vesicles are sufficient to mediate morphogenesis but we do not favor this explanation. We did consider the possibility the ectopic optic cup might recruit mesenchymal cells as the eye emits a diffusible signal that attracts cranial neural crest during its development (Langenberg et al., 2008; Kish et al., 2011). However, in only one relatively rostrally located transplant (of five transplants assessed) did we see recruitment of host neural crest cells to the ectopic optic cup. Consequently, despite severely compromised POM, ectopic optic vesicles form normal optic cups.

Several factors likely contribute to differences in severity of phenotypes when POM is disrupted *in vivo* and when eyes are grown ectopically or *in vitro*. First, genes required for POM development frequently have additional roles in other tissues and these other roles may contribute to the severity of eye phenotypes when gene function is disrupted. Second, we suggest that eye formation *in vivo* is both promoted and constrained by the environment in which optic cup morphogenesis normally occurs. In the vicinity of the forming eyes there are many contemporaneous and precisely coordinated developmental events occurring and the developing eye may have limited capability to cope with environmental disruption. For instance, the presence of mis-positioned, dying or abnormal POM may have much more severe consequences on eye morphogenesis than depletion/absence of such cells.

In summary, our study shows that ectopic optic vesicles show remarkably normally morphogenesis but fail to fuse the choroid fissure. This observation is consistent with the possibility that POM cells, which are throughout the fissure during the closure process, may play a role in fissure fusion. This could be, for instance, in degrading the basal laminae that subsequently enables fissure cells to remodel their apico-basal polarity and directly appose during the fusion process.

## Author contributions

GG and SW conceived and planned the study; GG, CS, and NB-L conducted experiments; GG and SW wrote the paper with comments and input from other co-authors.

### Conflict of interest statement

The authors declare that the research was conducted in the absence of any commercial or financial relationships that could be construed as a potential conflict of interest. The reviewer FC declared a past co-authorship with the authors GG and SW to the handling Editor.

## Supplementary material

Figure S1 Optic vesicle transplantation and POM migration. **(A)** Schematic showing the transplant procedure. Optic vesicles (ov) from Tg(*sox10:*GFP)^zf77^ embryos injected at the 1-cell stage with RNA encoding mRFP were transplanted at between 7 and 18 somites (ss, about 12–18 hpf) under the skin above the yolk of age-matched Tg(*sox10:*GFP)^zf77^ host embryos. The location of GFP-labeled neural crest cells posterior to the eye is shown in green. **(B)** Timeline showing timing of optic vesicle transplantation (12–18 hpf) and fixation (>100 hpf) relative to normal choroid fissure fusion (30–56 hpf). Green arrow indicates approximate onset of neural crest migration over the eye. **(C)** Combined brightfield and fluorescence image stills over 5.5 h of time lapse movies (Figure S1 and data not shown) from 9 to 18 ss showing migration of the two components of the periocular mesenchyme (POM) over the optic vesicle (outlined): cranial neural crest cells (upper panels; labeled by the *sox10*:GFP ^zf77^ transgene) and vascular mesodermal cells (lower panels; labeled by the *fli*:GFP ^y5^ transgene). Migration of the neural crest over the eye commences at ~9 ss (left side upper panel); successful transplants discussed in this study were performed between 12 ss (middle panels) and 18 ss (right side panels). **(D,E)** Whole mount embryos showing expression of the prospective retinal marker *rx2* in donor embryos fixed immediately post-surgery at ~12 ss. D shows one of 14/16 optic vesicles (ov) that were removed completely whereas E shows one of 2/16 transplants in which part of the vesicle was left behind; the host embryos from these two transplants were excluded from further analysis. **(F,G)** In order to verify that transplanted optic vesicle were not damaged by the transplant procedure, we performed control experiments in which the extirpated optic vesicle was transplanted orthotopically to its normal position and choroid fissure fusion assessed. These images shows ventral views of donor and a control host embryos from such control experiments in which an optic vesicle was transplanted back into its normal position showing that choroid fissure fusion (position marked by arrow) occurs in the transplanted orthotopic eye by 3 dpf (**G**, asterisk). **(H)** Variable amounts of transplanted neural crests, as labeled by the *sox10*:GFP transgene, were present in transplanted eyes. Compare control eyes with eyes transplanted with few (middle column) or more, but still reduced (right column), numbers of neural crest cells.

Figure S2 Failure of choroid fissure fusion in eyes derived from optic vesicles with reduced POM. **(A–F)** Methylene blue stained sagittal sections through ectopic eyes at 5 days post-fertilization. **(A)** Image of a section through a wild-type eye at 72 hpf. **(B,C)** Low resolution images of sections through an embryo with an ectopic eye on the yolk at the level of the back of lens **(B)** and deeper into the back of the retina **(C)** and higher resolution images through the ectopic eye showing that the retina is still open at its ventral pole **(D,F)** where the retinal lamination and retinal pigmented epithelium (RPE) are discontinuous. However, in the deeper layers, the outer nuclear layer is continuous across the presumed position of fissure fusion (arrow in **F**). **(D,E)** Arrows in **(D,E)** indicate the choroid fissure. SC, spinal cord; EE, ectopic eye; RGC, retinal ganglion layer; ONL, outer nuclear layer. Scale bars 50 μM.

Movie S1 Loss of α-catenin-labeled adherens junctions from the fusing choroid fissure. Time lapse movie of the choroid fissure from a Tg(*ctnna*:citrine)^ct3aGT^ embryo from 38 to 48 hpf. Maximum projection of 10 Z-sections per frame with an interval between frames of 10 min.

Movie S2 Some cells lining the choroid fissure can move toward the inner surface of the retina. Time lapse movie from 38 to 50 hpf of the choroid fissure in an embryo injected with RNA encoding H2B:RFP and Pard3:GFP. Individual nuclei are manually tracked showing that as Pard3 labeling retracts toward the outer retina, some nuclei of cells initially lining the fissure move toward the inner neural retina. The white dots disappear when we could no longer track the nuclei with confidence.

Movie S3 Cells lining the choroid fissure can show protrusive activity. Time-lapse movie of the choroid fissure in which all nuclei are labeled with nuclear-localized GFP (green) and transplanted cells are labeled with membrane targeted RFP (magenta). Z projections of sections covering 6 μm with an interval between frames of 10 min.

Movie S4 Cells lining the choroid fissure cells are proliferative. Time-lapse movie of the ventral eye in which all cells express membrane targeted RFP and one cell in the fissure margin expressing cytoplasmic GFP undergoes cell division. Z projections of sections covering 6 μm with an interval between frames of 10 min.

Movie S5 Periocular mesenchyme cells are actively motile in the choroid fissure. Time-lapse movie of an eye with all cell membranes labeled by the Tg(β-actin:HRAS-EGFP)^vu119^ transgene. Z projections of sections covering 18 μm with an interval between frames of 10 min.

Movie S6 The hyaloid artery is displaced superficially during choroid fissure fusion. Time-lapse movie of an eye with cell membranes labeled by mGFP injection. Z projections of sections covering 30 μm with an interval between frames of 10 min. Lateral view (rotated to look into the fissure) of the hyaloid vasculature.

Movie S7 Transplanted optic vesicles are depleted of neural crest. Time-lapse movie of Tg(*sox10:*GFP)^zf77^ neural crest cells (green) around the transplanted optic vesicle labeled in red (mRFP-positive), showing the presence of few neural crest cells. The olfactory placode (to the left in the movie) has also been transplanted. Z projections of sections covering 20 μm with an interval between frames of 15 min over a 12 h period from 20 to 32 hpf.

Movie S8 Recruitment of host neural crest cells to a transplanted optic vesicle. Time-lapse movie of Tg(*sox10:*GFP)^zf77^ cells (from the host) approaching from the right and interacting with a transplanted optic vesicle (the transplanted optic vesicle is visible in red; mRFP-positive) on the yolk. Z projections of sections covering 20 μm with an interval between frames of 15 min over a 12 h period from 20 to 32 hpf. The incoming cells appear somewhat differentiated and may be melanoblasts.
