# Supplementary figures and images for: Cell Behaviors during Closure of the Choroid Fissure in the Developing Eye
Source: Front Cell Neurosci. 2018 Feb 20;12:42. doi: 10.3389/fncel.2018.00042 (PMC5826230; doi:10.3389/fncel.2018.00042)

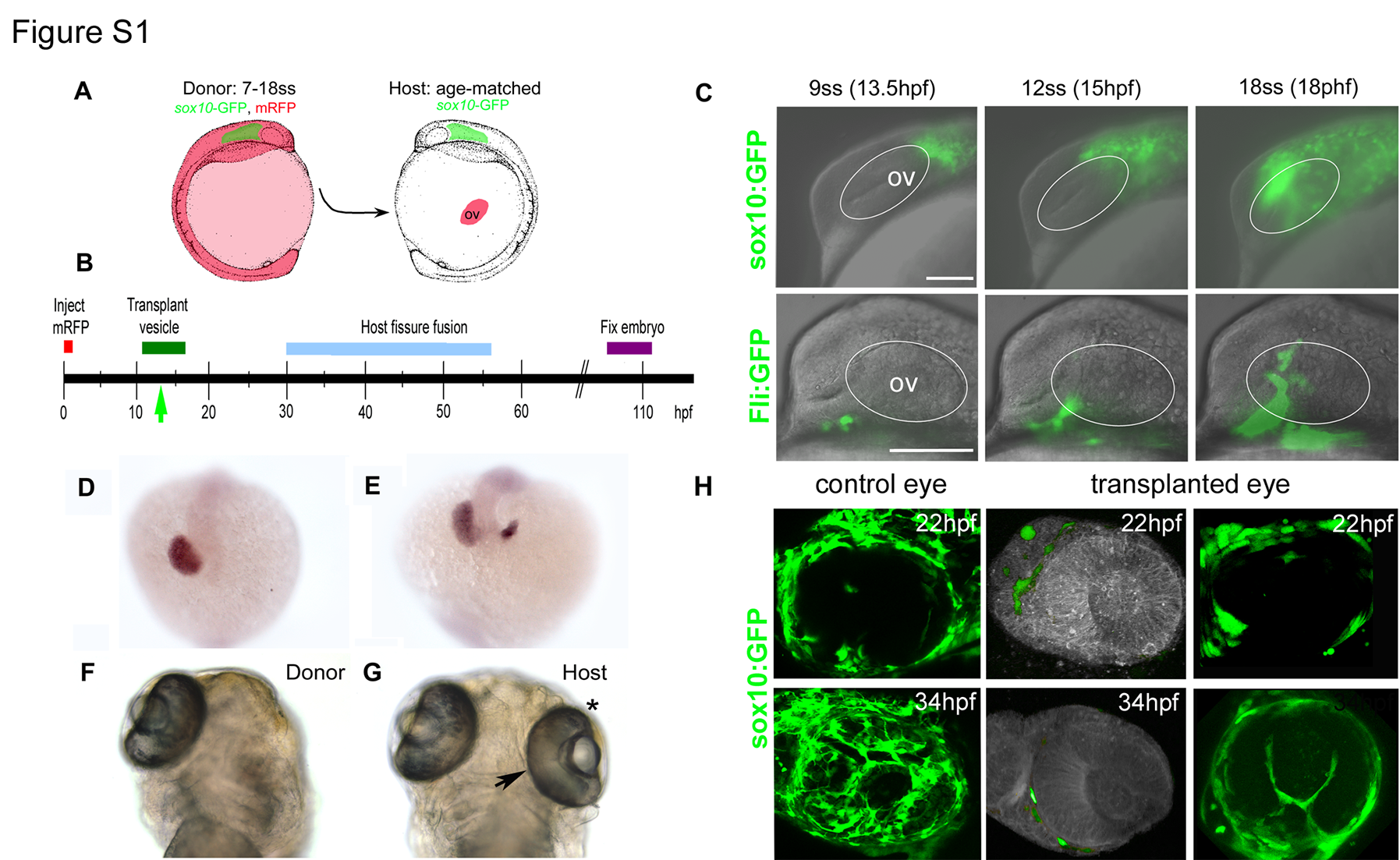

Supplement: Figure S1 — Optic vesicle transplantation and POM migration. (A) Schematic showing the transplant procedure. Optic vesicles (ov) from Tg(sox10:GFP)zf77 embryos injected at the 1-cell stage with RNA encoding mRFP were transplanted at between 7 and 18 somites (ss, about 12–18 hpf) under the skin above the yolk of age-matched Tg(sox10:GFP)zf77 host embryos. The location of GFP-labeled neural crest cells posterior to the eye is shown in green. (B) Timeline showing timing of optic vesicle transplantation (12–18 hpf) and fixation (>100 hpf) relative to normal choroid fissure fusion (30–56 hpf). Green arrow indicates approximate onset of neural crest migration over the eye. (C) Combined brightfield and fluorescence image stills over 5.5 h of time lapse movies (Figure S1 and data not shown) from 9 to 18 ss showing migration of the two components of the periocular mesenchyme (POM) over the optic vesicle (outlined): cranial neural crest cells (upper panels; labeled by the sox10:GFP zf77 transgene) and vascular mesodermal cells (lower panels; labeled by the fli:GFP y5 transgene). Migration of the neural crest over the eye commences at ~9 ss (left side upper panel); successful transplants discussed in this study were performed between 12 ss (middle panels) and 18 ss (right side panels). (D,E) Whole mount embryos showing expression of the prospective retinal marker rx2 in donor embryos fixed immediately post-surgery at ~12 ss. D shows one of 14/16 optic vesicles (ov) that were removed completely whereas E shows one of 2/16 transplants in which part of the vesicle was left behind; the host embryos from these two transplants were excluded from further analysis. (F,G) In order to verify that transplanted optic vesicle were not damaged by the transplant procedure, we performed control experiments in which the extirpated optic vesicle was transplanted orthotopically to its normal position and choroid fissure fusion assessed. These images shows ventral views of donor and a control host embryos fr [file Image1.TIF]

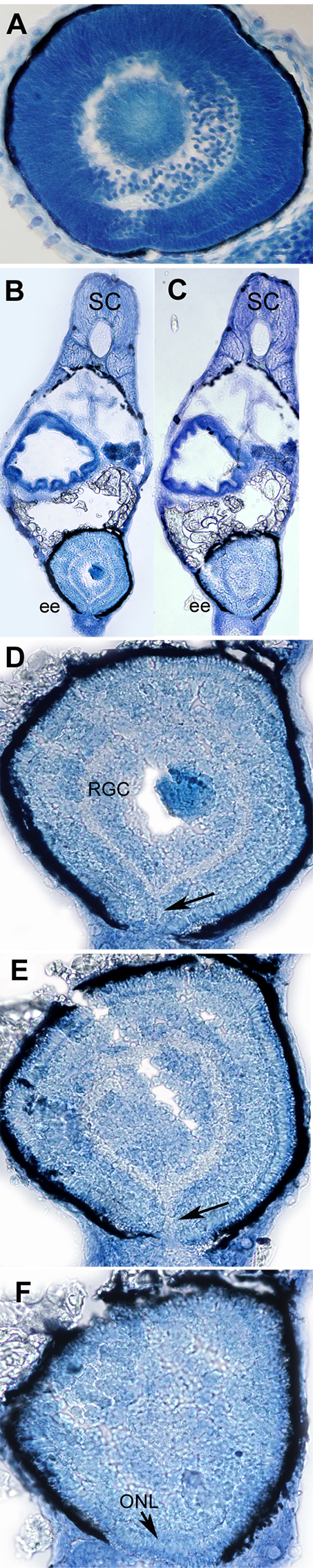

Supplement: Figure S2 — Failure of choroid fissure fusion in eyes derived from optic vesicles with reduced POM. (A–F) Methylene blue stained sagittal sections through ectopic eyes at 5 days post-fertilization. (A) Image of a section through a wild-type eye at 72 hpf. (B,C) Low resolution images of sections through an embryo with an ectopic eye on the yolk at the level of the back of lens (B) and deeper into the back of the retina (C) and higher resolution images through the ectopic eye showing that the retina is still open at its ventral pole (D,F) where the retinal lamination and retinal pigmented epithelium (RPE) are discontinuous. However, in the deeper layers, the outer nuclear layer is continuous across the presumed position of fissure fusion (arrow in F). (D,E) Arrows in (D,E) indicate the choroid fissure. SC, spinal cord; EE, ectopic eye; RGC, retinal ganglion layer; ONL, outer nuclear layer. Scale bars 50 μM. [file Image2.TIF]
